# 

**Published:** 1899-01-14

**Authors:** 


**Notices of Births, Deaths, and Marriages** are inserted in this column at a charge of 2s. 6d. for an announcement not exceeding four lines.

### NOTICE TO CORRESPONDENTS.

All MS., Letters, Books for Review, and other matters intended for the Editor should be addressed The Editor, "The Hospital," The Hospital Building, 28 & 29, Southampton Street, Strand, W.C.

The Editor cannot undertake to return rejected MS., even when accompanied by stamped directed envelope.

**Notice to Advertisers and Subscribers.**

All Advertisements, Orders for Copies of The Hospital, and Business Communications should be addressed to **THE MANAGER (Not to the Editor),** The Hospital, The Hospital Building, 28 & 29, Southampton Street, Strand, London, W.C.

The Cost of Subscription, post free, is as follows:—Per week, 2½d.; per quarter, 2s. 8d.; per half-year, 5s. 3d.; per annum, 10s. 6d. Foreign:—Per annum, 15s. 2d., payable, in all cases, in advance.

**NOTICE.—Vol. XXIV. of "The Hospital" (April, 1898, to September, 1898), in handsome cloth binding, is now on sale at the Office of this Journal, price 7s. 6d. Cases for binding the Numbers contained in this Volume 1s. 6d. Index to Vol. XXIV., 2½d., post free.**

**The Monthly Part for January is now ready. Price 6d., by post 8d.**

### BOOKS RECEIVED.

THE SCIENTIFIC PRESS.

"George Harley, F.R.S. The Life of a London Physician." By his daughter, Mrs. Alec Tweedie.

"Cleanliness in Children." By Rose Petty, Associate of the Sanitary Institute.

JOHN WRIGHT AND CO.

"Treatment of Disease by Physical Methods." By Thomas Stretch Dowse, M.D.

SWAN SONNENSCHEIN AND CO.

"Willie. A Story of a Children's Hospital." By M. Calderford.

WILLIAM BLACKWOOD AND SONS.

"The Good Regent." By Professor Sir T. Grainger Stewart, M.D., LL.D.

JAMES G. BISSET.

"A Handbook of Midwifery." Specially designed for the use of candidates for the certificate of the Obstetrical Society of London. By Alfred S. Gubb, M.D.

WARD, LOCK, AND CO.

"Book of Dogs."

THE BRITISH AND COLONIAL DRUGGIST.

"The British and Colonial Druggist Diary, 1899."

### THE STUDENT OF MEDICINE.

### APPOINTMENTS.

E. L. ANDERSON, L.S.A.Lond., appointed Assistant Medical Officer to the Mill Road Infirmary of the West Derby Union, Liverpool. G. Arkle, L.S.A., appointed Medical Officer for the Epileptic Institution at Belmont Grove of the West Derby Union. W. Barter, M.D., M.Ch., &c., appointed Medical Officer to the employés of the London District, Great Central Railway. J. C. Briscoe, M.R.C.S., L.R.C.P.Lond., appointed House Physician to King's College Hospital. C. P. Burd, M.R.C.S., L.R.C.P., appointed Assistant House Surgeon to the Salop Infirmary, Shrewsbury. P. C. Colls, M.R.C.S.Eng., L.R.C.P.Lond., appointed House Surgeon to King's College Hospital. R. Davies, M.D., Ch.M.Edin, M.R.C.S.Eng., L.R.C.P.Lond., appointed Medical Officer to the Workhouse of the Cheltenham Union. P. Furnivall, F.R.C.S.Eng., appointed Assistant Surgeon to the London Hospital. G. Gibb, M.A., M.B., appointed Junior Physician to the Royal Hospital for Sick Children, Aberdeen. H. E. Gough, L.R.C.P.Lond., M.R.C.S.-Eng., appointed Medical Officer of Health to the Norwich Rural District. F. Greaves, M.R.C.S., L.R.C.P., appointed House Surgeon to the Derbyshire Royal Infirmary. G. S. Haynes, M.R.C.S., L.R.C.P., appointed House Physician to Addenbrooke's Hospital, Cambridge. F. S. D. Hogg, M.R.C.S., L.R.C.P.Lond., appointed Medical Officer for the Bradwell District of the Malden Union. M. Hutchinson, L.R.C.S., L.R.C.P.I., appointed Medical Officer for the Buckhurst Hill District of the Epping Union. P. Levick, B.A., M.B., B.C.-Cantab., appointed House Surgeon to King's College Hospital. C. T. Lewis, M.R.C.S.Eng., L.R.C.P.Lond., appointed House Accoucheur to King's College Hospital. S. F. Lynch, M.R.C.S.-Eng., L.R.C.P.Lond., appointed House Surgeon to King's College Hospital. G. R. MacGregor, M.D.Aberd., appointed Medical Officer of Health to the Bingley Urban District. C. R. S. Marshall, M.A.Cantab., M.B., B.Ch.Vict., appointed Professor of Materia Medica and Therapeutics in St. Andrews University. J. J. Mason, L.R.C.P., L.R.C.S.Edin., appointed Medical Officer for the Bollington District of the Macclesfield Union. J. C. Matthews, B.A.Cantab., L.S.A.Lond., appointed an Assistant Medical Officer to the Gold Coast (Yarkwa) Railway, W. Africa. G. E. May, M.R.C.S.Eng., L.R.C.P.Lond., appointed Medical Officer for the Third District of the Ware Union. W. C. C. Pakes, D.P.H.Camb., appointed Professor of Hygiene in the Bedford College for Women. A. Y. Richardson, M.B., B.S.-Dunelm., appointed Second Assistant Physician to the Suffolk County Asylum. S. Richmond, M.D.Edin., M.R.C.S.Eng., appointed Medical Officer of Health to the Dartford Rural District Council. C. D. D. Roberts, M.B.Lond., M.R.C.S.Eng., appointed Medical Officer for the Workhouse and the First District of the Dursley Union. G. A. Roberts, M.R.C.S.Eng., L.R.C.P.Lond., appointed Assistant House Physician to King's College Hospital. W. St. A. St. John, M.R.C.S., L.R.C.P., appointed House Physician to the Derbyshire Royal Infirmary. L. D. Saunders, M.R.C.S.Eng., L.R.C.P.Lond., appointed Assistant House Accoucheur to King's College Hospital. G. E. Scholefield, M.D.Edin., D.P.H.Vict., appointed Medical Officer of Health to the West Lancashire Rural District Council. T. L. Webster, M.R.C.S., L.R.C.P., appointed Assistant Medical Officer at the Walton Workhouse of the West Derby Union.

